# Hit detection in serial femtosecond crystallography using X-ray spectroscopy of plasma emission

**DOI:** 10.1107/S2052252517014154

**Published:** 2017-10-13

**Authors:** H. Olof Jönsson, Carl Caleman, Jakob Andreasson, Nicuşor Tîmneanu

**Affiliations:** a Department of Physics and Astronomy, Uppsala University, Box 516, SE-751 20 Uppsala, Sweden; b Center for Free-Electron Laser Science, Deutsches Elektronen-Synchrotron, Notkestraße 85, DE-226 07 Hamburg, Germany; c ELI Beamlines, Institute of Physics, Czech Academy of Science, Na Slovance 2, CZ-182 21 Prague, Czech Republic; d Condensed Matter Physics, Department of Physics, Chalmers University of Technology, SE-412 96, Göteborg, Sweden; e Department of Cell and Molecular Biology, Uppsala University, Box 596, SE-751 24 Uppsala, Sweden

**Keywords:** hit detection, plasma emission spectra, serial femtosecond crystallography, protein structure

## Abstract

Serial femtosecond crystallography is an emerging and promising method for determining protein structures, making use of the ultrafast and bright X-ray pulses from X-ray free-electron lasers. The upcoming X-ray laser sources will produce well above 1000 pulses per second and will pose a new challenge: how to quickly determine successful crystal hits and avoid a high-rate data deluge. Proposed here is a hit-finding scheme based on detecting photons from plasma emission after the sample has been intercepted by the X-ray laser. Plasma emission spectra are simulated for systems exposed to high-intensity femtosecond pulses, for both protein crystals and the liquid carrier systems that are used for sample delivery. The thermal radiation from the glowing plasma gives a strong background in the XUV region that depends on the intensity of the pulse, around the emission lines from light elements (carbon, nitrogen, oxygen). Sample hits can be reliably distinguished from the carrier liquid based on the characteristic emission lines from heavier elements present only in the sample, such as sulfur. For buffer systems with sulfur present, selenomethionine substitution is suggested, where the selenium emission lines could be used both as an indication of a hit and as an aid in phasing and structural reconstruction of the protein.

## Abstract 2

Plasma simulations of photon emission spectra from protein crystals in an X-ray free-electron laser pulse show that spectroscopy can be used as a valid hit-detection technique in high-throughput serial femtosecond crystallography experiments.

## 1. Introduction   

High-intensity X-ray free-electron lasers (XFELs) have recently paved the way for many pioneering studies in physics and biology. In structural biology in particular, they have enabled a new approach to the structural determination of proteins, called serial femtosecond crystallography (SFX). Traditional X-ray sources like synchrotrons have been extremely successful, and to date over 100 000 protein structures have been determined using X-ray methods and deposited in the Protein Data Bank (Westbrook *et al.*, 2002 ▸). However, the classical method requires large crystals of high quality in order to obtain efficient diffraction at atomic resolution while withstanding radiation damage. The SFX approach is suitable for proteins that are difficult to grow as large crystals or are too radiation-sensitive for conventional approaches (Schlichting, 2015 ▸; Chapman, 2017 ▸), and can even be used to unveil protein dynamics using time-resolved studies (Aquila *et al.*, 2012 ▸). Since the first breakthrough in 2011, this field has grown rapidly and so far more than 100 structures have been determined using this method.

SFX requires a large number of small crystals that are imaged individually by high-intensity X-ray pulses from XFELs, before being destroyed by the ionizing pulse. This is basically the concept of ‘diffraction before destruction’, where radiation damage is mitigated simply by out-running it with femtosecond X-ray pulses. A complete data set requires a large number of imaged crystals, which makes SFX a high-throughput technique with requirements on the XFEL sources to deliver X-ray pulses with a high repetition rate. The Linac Coherent Light Source (LCLS) has produced most of the SFX structures so far and has a repetition rate of 120 Hz, but future sources will go beyond this number. The upcoming European XFEL will have 27 000 pulses per second, with a bunch train structure where pulses arrive approximately 200 ns apart, and the planned upgrade to LCLS aims to reach 1 MHz. Laser-driven X-ray sources, like the European Light Infrastructure (ELI Beamlines) will have a repetition rate of 1 kHz. Presently, the available flux of these sources may not be sufficient for serial crystallography, but recent advances in driver-laser technology indicate possibilities of significantly increasing the conversion efficiency (Weisshaupt *et al.*, 2014 ▸) and integrated flux (Hädrich *et al.*, 2014 ▸) of laser-driven X-ray sources. If good diffraction data from every pulse were collected, a complete data set required to provide a three-dimensional structure would be obtained in just a few minutes.

These developments pose several challenges to the SFX community, for example the need for fast sample delivery to replenish the destroyed sample and a robust hit-detection mechanism. A high repetition rate will require a quick judgement of a valid sample hit on a pulse-to-pulse basis. The two-dimensional area detectors that are used to collect diffraction patterns are a reliable way of detecting a crystal hit, but they have a slow readout compared with the high repetition rate of future sources. Furthermore, if all produced data were to be saved, this would lead to a data deluge: collecting a typical 4 megapixel diffraction image in every single shot could easily lead to data rates of 100 GB s^−1^ (Maia & Hajdu, 2016 ▸). A lot of the recorded data may not contain a reliable sample hit and would thus be unusable; SFX experiments today show a large variability in the hit rate, between 0.1–10%.

The general goal is to reduce the quantity of data to a manageable level by providing reliable hit detection. An earlier suggestion was to use ion emission from the sample as an indicator of a valid hit, for example fast protons that escape from the Coulomb explosion of aerosolized samples (Andreasson *et al.*, 2014 ▸). This approach will work for aerosolized samples, but not for samples delivered in a liquid jet, since the liquid jet will always be exposed to X-rays and ions will constantly be emitted from the water. In this study, we investigate the possibility of utilizing photons from plasma emission in the interaction region as a way of distinguishing hits from non-hits. A ‘hit’ is defined in this work as the X-ray pulse hitting protein in the interaction region, giving the possibility of detecting diffracted X-rays on a detector. A ‘non-hit’ is consequently defined as having no protein in the interaction region at the moment when the X-ray pulse arrives.

To detect and differentiate the two cases, a difference in the physical processes is first required. We assume that a hit would contain material of different elemental composition to a non-hit, and therefore the photon spectra from the material would also differ. The question that will be investigated here is if the differences are reproducible under varying intensity. The possibility of interference from other material present in the beam due to buffers or sample-delivery methods will also be investigated.

**Figure 1:**
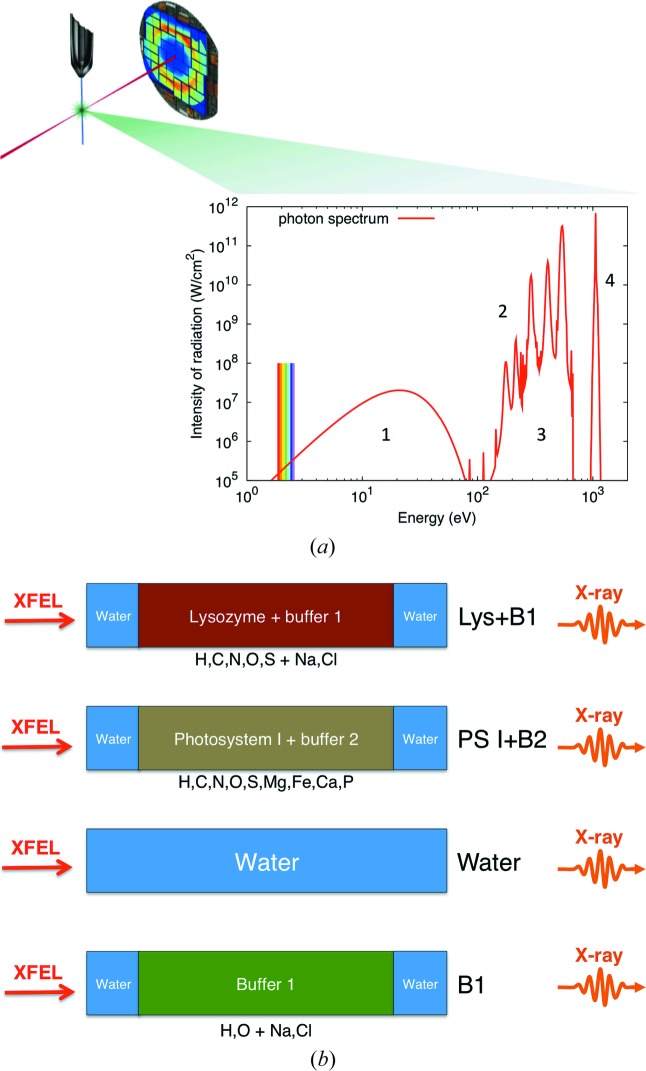
(*a*) Experimental sketch with the expected photon spectrum from plasma emission. An intense X-ray pulse hits the liquid jet that delivers the protein crystals and diffraction is recorded on an area detector. A photospectrometer records the plasma emission. The emitted photons could have several contributions, from (1) black body radiation, (2) characteristic line emission, (3) free–bound emissions and (4) scattering. The spectra are expected to be different for different samples. (*b*) The simulated sample geometries in this study. The samples have an external layer of 1 µm water sheaths, and inner parts with lysozyme, photosystem I, pure water and NaCl buffer. The simulations use one-dimensional geometry, with the XFEL pulse coming from the left and the emission spectra towards the spectrometer on the right.

The main idea is summarized in Fig. 1 ▸(*a*), showing a sketch of a standard SFX experiment, where the sample is delivered in a liquid jet and intercepted by an X-ray pulse which will diffract to produce a scattering pattern. Apart from the diffraction image, a plasma emission spectrum from the interaction region can be recorded on a photospectrometer, and several physical processes are expected to show up in the spectrum. The sample will ionize and turn into plasma, which will emit black body radiation (also called thermal radiation) depending on the equilibrium temperature, beyond the IR and optical regions, as shown explicitly in the figure. Characteristic line emissions, also known as fluorescence, from bound–bound and free–bound electronic transitions are expected in the plasma, and these depend directly on the composition of the sample. Finally, inelastically and elastically scattered photons will also be detected, with energies that depend on the incident photon energy. In this study, we simulate several sample compositions, including protein, buffers and water, as shown in Fig. 1 ▸(*b*).

It might also be useful to mention the framework of this work. The creation and diagnostics of solid-density plasmas with XFELs have been pioneered at LCLS (Vinko *et al.*, 2012 ▸), and a full experimental investigation of solid-density plasmas from biological systems is outside the scope of this article. Theoretical methods using non-local thermodynamic equilibrium (NLTE) population kinetics software codes are well developed and one particular area of application is in XFEL spectroscopy (Chung & Lee, 2009 ▸). X-ray spectroscopy has been used simultaneously with diffraction to study protein structure and can even give insight into the protein dynamics (Kern *et al.*, 2013 ▸). Standing on the shoulders of these giants, our aim is simply to give a blueprint for initial experiments that test hit detection in protein crystallography using plasma emission spectroscopy, and identify some of the challenges that lie ahead for high-throughput crystallography experiments at future XFEL sources.

## 2. Method   

The non-local thermodynamic equilibrium (NLTE) radiation-transfer plasma software code *Cretin* (Scott, 2001 ▸) was used to simulate X-ray interaction with the samples and obtain spectra for different samples. This code has previously been used successfully to simulate results from experiments where high photon fluence ionization quickly creates warm dense matter conditions in soft matter (Barty *et al.*, 2012 ▸). Agreement between simulation and experiment was shown with both high and low fluence using soft X-rays in condensed matter (Bergh *et al.*, 2008 ▸; Andreasson *et al.*, 2011 ▸; Rath *et al.*, 2014 ▸).

The code also tracks the time evolution of relevant properties, such as electronic states, transition rates, radiation spectra and temperatures (Caleman, Bergh *et al.*, 2011 ▸). A screened hydrogenic model was used for all elements in the sample composition. Double-core holes and multi-photon ionization processes were implemented in the simulation. *Cretin* models changes in the absorption cross section due to electron excitation and depletion of electronic states, as well as continuum lowering. The code treats secondary ionization processes such as electron–ion collisions and assumes instant thermalization of electrons, where electrons follow a Maxwellian energy distribution. The choice of electron–ion coupling coefficient will affect the dynamics of the atom and ion energies in the system. Here, the coefficient is calculated with Spitzer’s formula (Spitzer, 1956 ▸), using a Coulomb logarithm introduced by Gericke *et al.* (2002 ▸) for dense systems. Another effect of a dense system is a lowering of the continuum edges, here calculated by the Stewart–Pyatt formula (Stewart & Pyatt, 1966 ▸), a common approximation that has been tested against both experiment and more detailed models (Nantel *et al.*, 1998 ▸). Hydrodynamic expansion was also simulated and the expansion found was of the order of 0.1%, so it is not considered an important factor on femtosecond timescales.

For our studies, a scheme with a photon energy of 7000 eV (corresponding to a wavelength of 0.18 nm) and intensities ranging from 10^18^ to 10^21^ W cm^−2^ was used. Pulses were simulated as 20 fs duration, and the spectrum was collected for a total of 0.2 ps of simulation. For the energy spectrum of the emitted radiation, a large number of energy bins were placed on a logarithmic scale between 0.1 and 10 000 eV. Bins were reduced in size until no further effect on the simulation was seen. The energy bins are used to calculate the electronic state and the energy transfer between zones, as well as for the spectra for emitted radiation.

In our treatment of the sample, every zone/region is treated separately as a continuum with neutral net charge and mass conservation, but with radiation and heat transport occurring between neighbouring zones. While this geometry is un­suitable for very small samples or at the edges of a protein crystal where escaping electrons and ions must be considered (Caleman, Huldt *et al.*, 2011 ▸), the middle zones well describe the bulk properties of a crystal. The number of zones/regions can be chosen arbitrarily and is a trade-off between computational efficiency for a small number of zones and high accuracy with a large number of simulation zones.

As a model sample, five continuous one-dimensional zones were used. The simulated sample was considered to be 5 µm thick, which is far longer than the displacement distances that any ions will move within the 200 fs simulation. Fig. 1 ▸(*b*) shows in detail the four cases that were simulated: lysozyme embedded in a water jet (Lys+B1), photosystem I inside a water jet (PS I+B2), pure water, and 1.8 *M* NaCl buffer in water (B1). The geometry with an outer sheath of liquid corresponds to a double-flow focusing liquid jet, as has recently been used successfully to increase the performance of serial crystallography experiments (Oberthuer *et al.*, 2017 ▸). The atomic composition of each sample is presented in the Appendix[App appa]. Spectra from the outermost zone were studied, unless stated otherwise. The spectra are the sum of all radiation travelling in the direction opposite to the beam. Not only is radiation emitted in this direction considered, but absorption in layers on the way is also included. Changes in the absorption due to changes in the electronic state and temperatures are modelled for each time step. A time step of 50 attoseconds was used.

Our choice of simulated laser intensities represents the intensities that are available at the LCLS today on the various experimental stations, and which could be expected using the 0.1 µm diameter focus on the scientific instrument Single Particles, Clusters and Biomolecules (SPB) at the European XFEL (Mancuso, 2012 ▸). For example, the low end of our simulations, 10^18^ W cm^−2^, corresponds roughly to the intensity on the CXI end station with a micrometre focus, where many experiments have been performed to date (Boutet *et al.*, 2012 ▸; Redecke *et al.*, 2012 ▸; Kern *et al.*, 2014 ▸). The high end of our simulations, 10^21^ W cm^−2^, corresponds to intensities in the 100 nm focus (Nass *et al.*, 2015 ▸). Note also that, depending on the sample-delivery technique, the samples will be exposed to differing intensities on a shot-to-shot basis, as the protein crystals will stochastically end up in the spatial focus profile of the beam.

## 3. Results   

**Figure 2:**
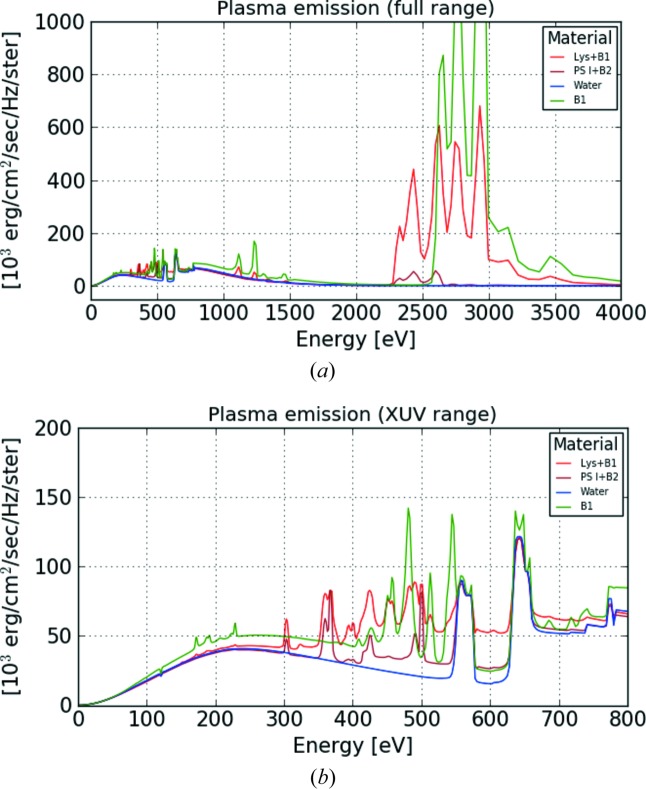
(*a*) Full spectra for the four different samples. The largest difference can be seen for lysozyme (Lys+B1) and buffer (B1), due to the strong chloride buffer that creates the largest difference from pure water at energies 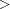
2.5 keV. At around 2.3 keV the sulfur, only present in photosystem I (PS I+B2), gives a signal while neither water nor buffer B1 emits anything. (*b*) Enlargement of the XUV region. Note that carbon and nitrogen will give signal above both water and buffer in the region 300–500 eV, but this signal is on top of the black body background. Simulations were done with 10^20^ W cm^−2^.

**Figure 3:**
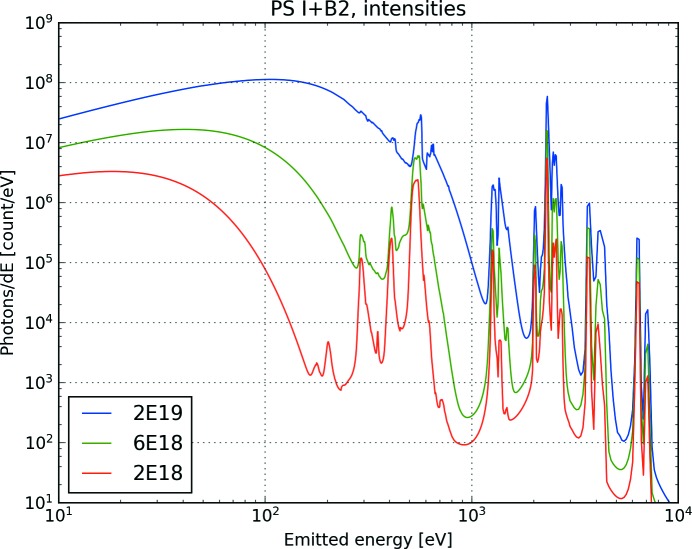
Spectra from photosystem I (PS I+B2) as a function of incident intensity. In the low-energy region the black body radiation will be strongly affected by an increase in the number of photons hitting the sample. At higher intensities the overall strength of the hit increases, and new features like line-broadening will appear. The electron temperatures at the end of the pulse are 13 eV (for 2 × 10^18^ W cm^−2^), 28 eV (for 6 × 10^18^ W cm^−2^) and 71 eV (for 2 × 10^19^ W cm^−2^).

Our simulations show rich spectra with many features (Fig. 2 ▸). The features can roughly be divided into two categories: a broad background, and specific lines from electronic transitions. The electronic transitions originate either from energy levels within the ions (so called bound–bound transitions), or into or from the continuum (free–bound transitions), and will be element-specific. The black body radiation is the contribution of the heated plasma, with temperatures reaching 100 eV for the highest simulated intensities, which causes a background for all of the spectra of the same order as the finer features that are the result of electronic transitions. This background is highly dependent on the incident intensity, which drives the final plasma temperature (Fig. 3 ▸).

Fig. 2 ▸ shows that protein samples will have a distinct signal from carbon and nitrogen (between 300 and 500 eV), which could in principle be used to differentiate from water. However, this signal is on top of a broad black body radiation background, which is strongly dependent on the incident intensity (Fig. 3 ▸). As the incident number of photons on the sample depends on where in the X-ray focus the sample has been intercepted, we expect there would be a large variation from shot to shot in the background radiation, which would have a negative impact on the reliability of a hit finder based on carbon or nitrogen lines. The region above 1 keV, where heavier elements have their emission lines (like Cl or S), provides a cleaner background which is not as strongly influenced by the warm dense plasma.

**Figure 4:**
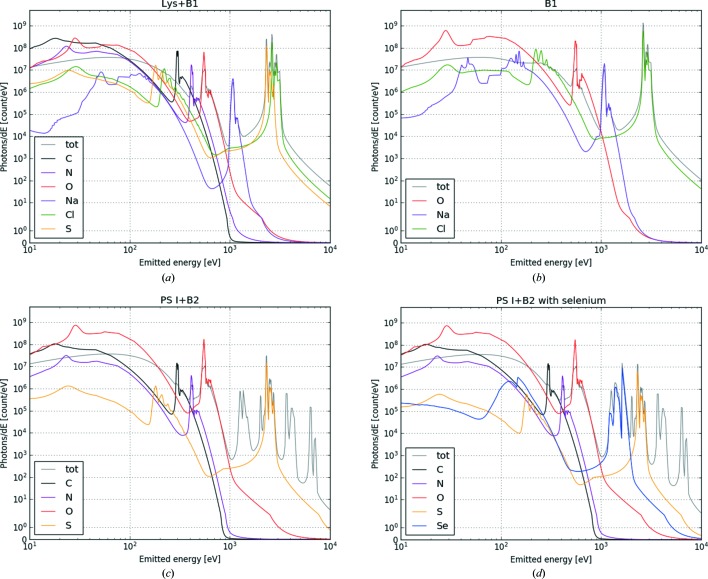
Total spectra from the sample and emission spectra for different elements. The element spectra are from the middle of the sample only. The total signal includes absorption effects from the sample as the signal goes through. Simulations were done with 10^19^ W cm^−2^. The samples are (*a*) lysozyme (Lys+B1), (*b*) NaCl buffer (B1), (*c*) photosystem I (PS I+B2) and (*d*) photosystem I where the methionine S atoms have been exchanged with selenium. Above 1 keV, the heavier elements have emission lines beyond the black body background (the temperature is the same for all ions). The selenium substitution shows the possibility of biochemically adding new spectral features, with selenium (in blue) different from sulfur (in yellow). Note that the scale is in emitted photons per energy unit, to emphasize that the emitted number of photons at high energy could be considerable.

The plasma code can simulate the emission spectra for the individual atomic species in the sample, and these are shown in Fig. 4 ▸ together with the total spectrum. The NLTE code uses a two-temperature model, one for electrons and one for ions, where all ions have the same temperature. This can be seen in the figure, in the black body radiation background that corresponds to the same temperature for all ions. The figure also shows a drastic reduction in the background above 1 keV, where the *K*α_1_ and *K*α_2_ lines for heavier elements (Cl, S, Se) can be observed. We have also simulated a special case for photosystem I, where some S atoms have been replaced by Se atoms, inspired by a commonly used method for phasing in crystallography called selenomethionine substitution. The Se spectrum has characteristic emission and could allow for easier hit finding. This is seen in Fig. 4 ▸, where the selenium *L*α_1,2_ and *L*β_1_ transitions are at around 1380 and 1420 eV, respectively, in a region otherwise empty of features.

**Figure 5:**
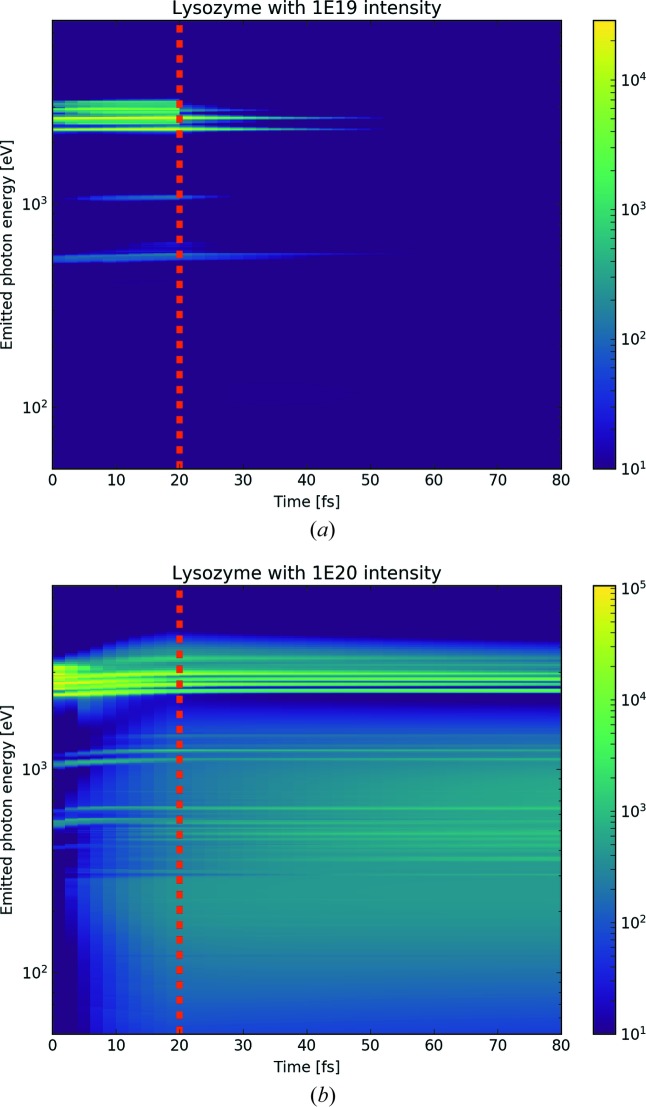
Emission spectra from the lysozyme sample (Lys+B1) as a function of time for two intensities, (*a*) 10^19^ W cm^−2^ and (*b*) 10^20^ W cm^−2^. The colour scale indicates emission in erg cm^−2^ sec^−1^ Hz^−1^ sterad^−1^. The dotted line at 20 fs shows the end of the X-ray pulse. Depending on the intensity, the plasma will continue to glow after the pulse, both in the XUV region and in the *K*α region, due to continuous ionization from the hot electron gas.

Another observation can be made when the spectra are plotted as a function of time, as seen in Fig. 5 ▸. The background radiation builds up during the pulse, as the electron gas gets hotter, but it will also continue to glow after the pulse has ended, until the plasma has expanded and cooled down. Furthermore, the characteristic emission lines which are present during the pulse might also continue to be present after the pulse. During the pulse, the *K*α fluorescence is a result of a core–hole relaxation originating from direct photo-ionization, while after the pulse the core–hole is produced from electron collisions in the hot plasma. This process will be proportional to the X-ray intensity. We note also that high X-ray intensity would also lead to a shift of the emission lines as well as broadening, a direct result of the continuum-lowering model.

## 4. Discussion and conclusions   

The premise of our study is that it should be possible to distinguish quickly between hits and non-hits in an XFEL diffraction experiment. As the repetition rate of ultrafast X-ray sources increases, the size of the data set collected will increase as well. This calls for the development of online methods that discriminate between shots with and without a sample particle in the interaction region. We argue that a spectroscopic method using features in emitted photon spectra might be extremely useful to determine whether a certain sample in a liquid jet environment has been hit by the XFEL pulse, and maybe even to classify hits from different heterogeneous samples.

The basis of diffractive imaging with ultrafast pulses is ‘diffraction before destruction’. The diffracted X-ray photons carry information about the structure and its dynamics only during the pulse, which should be as short as possible to ensure that atomic motion does not limit the resolution. In contrast, using photons from plasma emission is more akin to ‘spectrum during destruction’. Plasma dynamics in sub-micrometre samples will happen on a picosecond time scale, during which the plasma will continue to emit both thermal radiation and specific emission lines, if the electron temperature is high enough to ensure ionization of deep electronic levels.

In this work we have shown that a qualitative difference can be observed between the spectrum from a sample, in our examples lysozyme and photosystem I, and the spectrum from the carrier liquid, such as water or a sodium chloride buffer. It becomes apparent that using the absolute intensity of a spectral feature or peak at a fixed energy is an uncertain indicator of a hit, as this can have a strong dependence on the incident photon intensity. Not only do the absolute levels change with intensity, but also different physical processes come into play and the thermal background will give different contributions. If one were to use a photospectrometer in the 100–1000 eV region to distinguish between hits and non-hits in the intensity range of the present study, then one would need to find ways to identify the carbon and nitrogen with high energy resolution, above an unstable background that can vary from shot to shot. The advantage of an XUV spectrometer is that it could provide a good shot-to-shot measurement of the temperature of the plasma, and consequently additional information on the intensity of each hit.

Comparing protein crystals in buffer systems with clean water offers many possibilities for tests. In reality, the water jet delivery system may not be just pure water, as buffers will always be present even if a protein crystal is not. Here more complex tests must be used, and the buffer composition will play a part and needs to be studied in detail. When sulfur is present in the sample, as is the case for almost all proteins, any photon emitted in the 2.0–2.3 keV region is a clear indicator of a hit compared with most buffers without sulfur. This region is also beyond the thermal radiation background, and could perhaps even allow for an absolute photon count method for hit finding, for example using photodiodes or microchannel plate detectors. Such an approach would also have the advantage of providing a fast readout and quick judgement of crystal hits, and could in principle be implemented as part of the data collection. The speed of an automated process is not straightforward to estimate, as it depends on the spectrometer. We assume that collecting a photon spectrum would be considerably faster than collecting a two-dimensional image with a CCD camera and selecting a hit by selecting for Bragg peaks.

From a spectroscopy point of view, it would be difficult to distinguish between proteins in solution and proteins in crystals. It is here assumed that the protein concentration in the surrounding liquid is very low and will give a weak emission. Our experience with lysozyme crystals shows that the protein concentration in the solution surrounding the crystal is very low (0.3% of the original concentration). Under such conditions, we expect the photon emission from the liquid to be much weaker than the signal from the crystal. In the cases where this is not true, one can have two approaches. One would be to exchange the buffer just prior to sample delivery. The other is to acknowledge that the high repetition rate must lead to data being thrown away, and that protein in the interaction region can be used as a first requirement for keeping a specific frame from the detector. In the latter case, additional criteria requiring a significantly longer analysis time, such as the presence of a Bragg signal on the detector, could also be used.

A wide variety of buffers are used in protein crystallography and in SFX. Some buffers could have much higher concentrations than we simulated here, for instance the use of 3.3 *M* ammonium sulfate acid has been reported in the literature (Nass *et al.*, 2015 ▸), and simulations with such specific conditions would need to be performed for hit finding. Other crystallographic techniques use selenomethionine substitution, or phasing agents such as I3C containing iodine, and the presence of heavy atoms could provide a unique fingerprint in the emission spectrum above 1 keV. The strength of our proposed method is that basic knowledge of the composition of crystals, combined with plasma simulations and an implementation of a photon spectrometer, should be enough to provide a platform for promising hit finding.

## Figures and Tables

**Table 1:** Sample densities (g cm^−3^)

Lysozyme	Photosystem I	Water	Buffer	Lysozyme
Lys + B1	PS I + B2		B1	Pure protein
1.26	1.08	1.00	1.08	1.35

## Appendix A Sample composition

The densities used in the simulations are shown in Table 1 ▸. The atomic composition of the samples is given as the number of atoms/stoichiometric ratio:

(i) Buffer B1: 600 H, 300 O, 10 Na, 10 Cl.

(ii) Lys + B1: consists of lysozyme protein 2/3 + buffer 1/3 (*v*/*v*), where lysozyme contains 959 H, 613 C, 193 N, O 185, 10 S, and buffer B1 is as shown in (i) above (Lomb *et al.*, 2011 ▸).

(iii) PS I + B2: consists of photosystem I including special additive buffer, with 141 000 H, 16 900 C, 3310 N, 57 300 O, 89 S, 96 Mg, 12 Fe, 1 Ca, 3 P (Barty *et al.*, 2012 ▸).

(iv) PS I + B2 with selenium: similar to (iii) above but with methionine S atoms replaced with Se, 141 000 H, 16 900 C, 3310 N, 57 300 O, 39 S, 50 Se, 96 Mg, 12 Fe, 1 Ca, 3 P.
